# A new cyst-forming nematode, *Cactodera tianzhuensis* n. sp. (Nematoda:Heteroderinae) from *Polygonum viviparum* in China with a key to the Genus *Cactodera*

**DOI:** 10.21307/jofnem-2021-029

**Published:** 2021-03-24

**Authors:** Wenhao Li, Huixia Li, Chunhui Ni, Mingming Shi, Xuejuan Wei, Yonggang Liu, Yiwen Zhang, Deliang Peng

**Affiliations:** 1 College of Plant Protection, Gansu Agricultural University/Biocontrol Engineering Laboratory of Crop Diseases and Pests of Gansu Province, Lanzhou 730070, Gansu Province, China; 2 Institute of Plant Protection, Gansu Academy of Agricultural Sciences, Lanzhou 730070, Gansu Province, China; 3 State Key Laboratory for Biology of Plant Diseases and Insect Pests, Institute of Plant Protection, Chinese Academy of Agricultural Sciences, Beijing 100193, China

**Keywords:** *Cactodera*, Morphology, Morphometrics, Phylogeny, New species, Taxonomy

## Abstract

A new cyst-forming nematode, *Cactodera tianzhuensis* n. sp. was isolated from the rhizosphere soil of *Polygonum viviparum* L. in Tianzhu county, China. Morphologically, the new species is characterized by lemon-shaped or rounded cysts that have protruding necks and vulval cones. The vulval cone of the new species appeared to be circumfenestrate without bullae and underbridge, vulval denticle present and anus distinct. Second-stage juveniles are vermiform, stylet well-developed with the rounded stylet knobs to slightly concave anteriorly. Lateral field with four incisures. Tail gradually tapering to a finely rounded terminus with a length of *ca* 54 (47–59) µm, outline of hyaline portion is V-shaped or U-shaped. Egg shells without visible markings or punctations. The phylogenetic analyses based on ITS-rDNA, D2-D3 of 28S-rDNA clearly revealed that the new species formed a separate clade from other *Cactodera* species, which further support the unique status of *C. tianzhuensis* n. sp. Therefore, it is described herein as a new species of the genus *Cactodera*.

*Cactodera* is a cyst-forming nematode genus of the Heteroderidae erected by Krall and Krall (1978) and the type species is cactus cyst nematode, *Cactodera cacti* (Filipjev and Schuurmans Stekhoven, 1941) Krall and Krall, 1978, which is distributed worldwide and mainly damaged plants of the family Cactaceae grown in glasshouse as ornamental (Skantar et al., 2019). *Cactodera* spp. are mainly characterized based on vulval region fenestration, bullae and underbridge absent or present in cyst, the length of stylet, tail and hyaline tail in second-stage juvenile, and the surface differentiation in eggs (Subbotin et al., 2010). However, traditional identification of cyst forming nematode based on morphology is imprecise and time-consuming to separate the related species. During the past 30 years, molecular data, including ITS-rDNA, D2-D3 region of 28S-rDNA, are more accurate tool for identification of cyst-forming nematode species. Sequence analysis of the ITS-rDNA and the D2-D3 region of 28S-rDNA of unknown species is sufficient to study the phylogenetic relationship and identify cyst-forming nematode species (Maafi et al., 2003; Subbotin et al., 2001, 2006).

Up to now, the genus *Cactodera* contains 16 valid species and mostly parasites plants of Amaranthaceae, Cactaceae, Chenopodiae, and Polygonaceae in different regions (Escobar-Avila et al., 2020; Feng et al., 2018; Subbotin et al., 2010). To date, very little is known about the occurrence and distribution of *Cactodera* nematode and only three species of *Cactodera* have been reported in China: *C. cacti* (Filipjev and Schuurmans Stekhoven, 1941) Krall and Krall, 1978 parasitizing the roots of *Opuntia dillenii* (Pan et al., 1997) and *Hylocereus undatu* (Duan et al., 2012) in Fujian and Liaoning province, respectively; *C. thornei* (Golden and Raski, 1977) Mulvey and Golden, 1983 was found in cereal fields in Qinghai province (Peng and Vovlas, 1994); *C. chenopodiae* Feng et al., 2018 was described as a new cyst-forming nematode in the genus *Cactodera* parasitizing on *Chenopodium album* in Liaoning province (Feng et al., 2018).

During 2019–2020, a population of cyst-forming nematodes was found from the rhizosphere of *Polygonum viviparum* L. in Tianzhu county of Gansu Province, China, based on morphological, morphometric and molecular analyses. Its characters were then compared with all the related species of the genus *Cactodera.* This population is described herein as *Cactodera tianzhuensis* n. sp. due to its unique characters. To help identify the species in the genus *Cactodera*, a key to *Cactodera* species is presented.

## Materials and methods

### Nematode extraction and morphological characterization

Cysts, second-stage juveniles of new species were extracted from roots and soil samples of the host plant, *Polygonum viviparum*, in Tianzhu county, Gansu Province, China, using standard centrifugal flotation (Jenkins, 1964) and Fenwick method (Fenwick, 1940), respectively. Males were not found. For morphometric studies, second-stage juveniles were killed by gentle heating, fixed in TAF solution (formalin:triethanolamine:water = 7:2:91), and processed to ethanol-glycerin dehydration according to Seinhorst (1959) and mounted on permanent slides. For observation of vulval cones, cysts were soaked in water for several hours and dissected, the vulval cone can be bleached for 5 min in H_2_O_2_ and dehydration in different gradient alcohol, the last mounted in glycerin jelly on glass slide (Subbotin et al., 2010). Light micrographs and measurements were conducted on mounted specimens using Zeiss Axio Scope A1 (Zeiss, Jena, Germany) equipped with an AxioCam 105 color camera, drawings were accomplished using a drawing tube attached to Nikon YS 100 (Nikon, Tokyo, Japan) and improved using the software Adobe illustrator CS6 x64 Version 13.0.1.

### Molecular analyses

DNA was extracted from single cyst (containing J2s and eggs) using Worm lysis buffer [50 mM KCl, 10 mM Tris (pH = 8.3), 2.5 mM MgCl_2_, 0.45% Nonidet P-40 and 0.45% Tween 20] in conjunction with proteinase K (1 mg/ml). Two sets of primers (synthesized by Tsingke Biotech Co. Ltd, Xi’an, China) were used in the PCR analyses to amplify sequences of the ITS and D2-D3 segments of 28 S. The ITS region was amplified with TW81 (5′–GTTTCCGTAGGTGAACCTGC–3′) and AB28 (5′–ATATGCTTAAGTTCAGCGGGT–3′) (Maafi et al., 2003). The 28 S D2-D3 region was amplified with the D2A (5′–ACAAGTACCGTGAGGGAAAGTTG–3′) and D3B (5′–TCGGAAGGAACCAGCTACTA–3′) (De Ley et al., 2005; Ye et al., 2007). Detailed protocols for DNA extraction, PCR conditions used in this study were as described by Munawar et al. (2018), Maafi et al. (2003), and Subbotin et al. (2001). PCR products were separated on 1% agarose gels and visualized by staining with ethidium bromide. PCR products of sufficiently high quality were purified for cloning and sequencing by Tsingke Biotech Co. Ltd., Xi’an, China. The PCR products were purified by the Tiangen Gel Extraction Kit (Tiangen Biotech Co. Ltd., Beijing, China), cloned into pMD18-T vectors and transformed into DH5α competent cell, and then sequenced by Tsingke Biotech Co. Ltd (Xi’an, China).

### Sequence alignment and phylogenetic analysis

The newly obtained sequences for ITS-rDNA and D2-D3 of 28S-rDNA region were compared with known sequences of other related species on GenBank using BlastN homology search program. Outgroup taxa for phylogenetic analyses were selected based on the previously published studies (Cid Del Prado and Subbotin, 2014; Escobar-Avila et al., 2020; Feng et al., 2018; Soto et al., 2003; Subbotin et al., 2006, 2017). All the selected sequences were aligned by MAFFT (Standley, 2013) with default parameters and edited using Gblock (Castresana, 2000). Phylogenetic analysis of ITS-rDNA and D2-D3 of 28S-rDNA region were based on Bayesian inference (BI) using MrBayes 3.2.6 (Huelsenbeck and Ronquist, 2001). The GTR + I + G model was selected as the best-fit model of DNA evolution using MrModeltest version 2.3 (Nylander, 2004) according to the Akaike Information Criterion (AIC). BI analysis for each gene was initiated with a random starting tree and run with four Markov chains (three heated and one cold) for 1,000,000 generations. The Markov chains were sampled at intervals of 100 generations and the burn-in value was 25%. Two runs were performed for each analysis. After discarding burn-in samples, the remaining samples were used to generate a 50% majority rule consensus tree. Posterior probabilities (PP) were given on appropriate clades. The phylogenetic consensus trees were visualized using the software FigTree v.1.4.3 (http://tree.bio.ed.ac.uk/ software/figtree/) (Rambaut, 2016).

## Results

### Systematics

*Cactodera tianzhuensis* n. sp.

http://zoobank.org/urn:lsid:zoobank.org:act: 09128DB0-6CC4-4F9C-B084-4CE1B9877E38.

**Figure 1:**
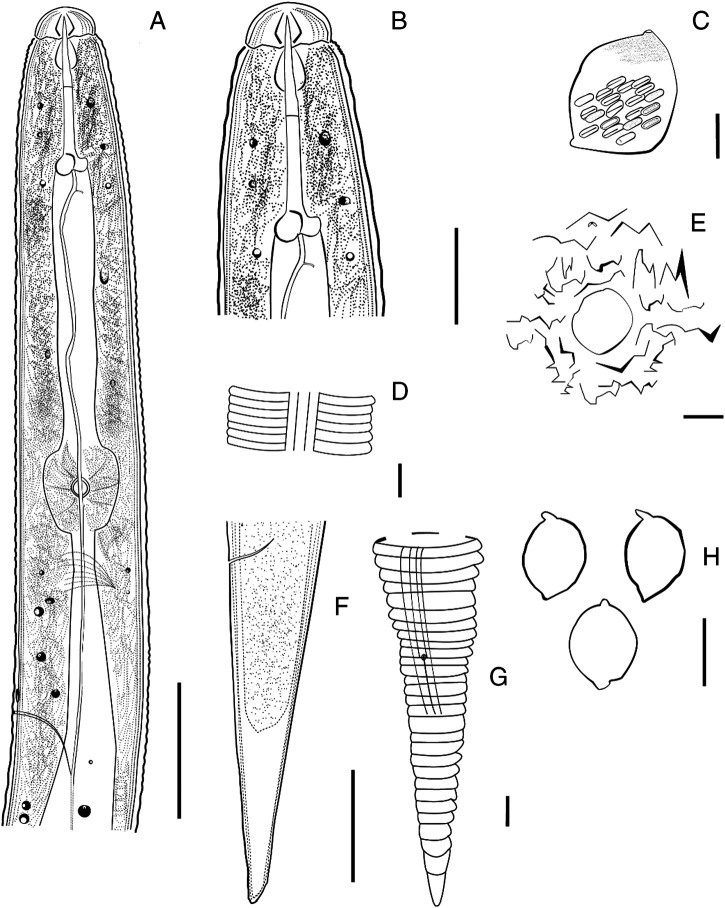
Line drawing of *C. tianzhuensis* n. sp. A: Anterior region of second-stage juvenile; B: Head of second-stage juvenile; C, H: Cyst and cysts. D: Lateral field; E: Fenestration in vulval cone; F, G: Tail of second-stage juvenile (Scale bar: H = 500 μm; C = 200 μm; A, F, E = 20 μm; B = 10 μm; D, G = 5 μm).

**Figure 2:**
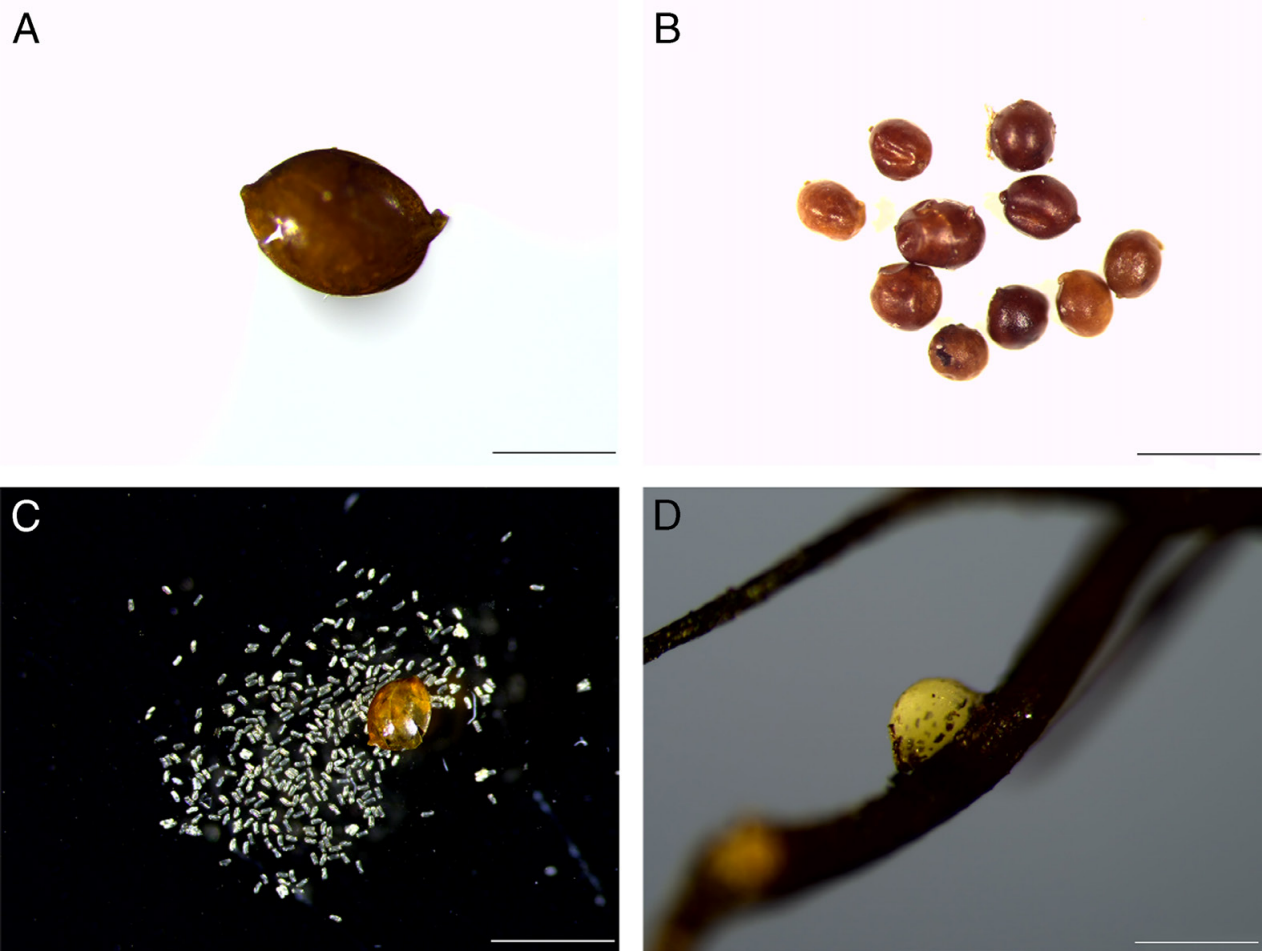
Light micrographs of *C. tianzhuensis* n. sp. A: Cyst; B: Cysts; C: Crush cyst; C: Female attached on the root (Scale bar: A, D = 500 μm; B, C = 1 mm).

**Figure 3:**
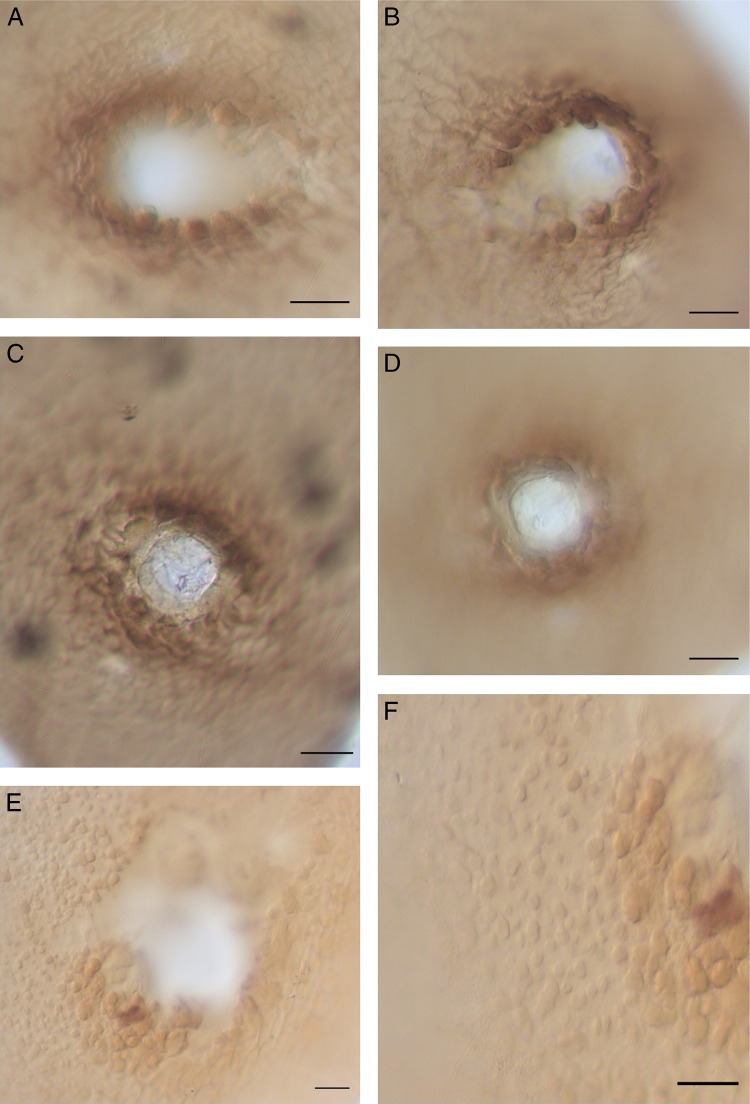
Vulval cones of *C. tianzhuensis* n. sp. A, C: Fenestration in vulval cone (inside); B, D: Fenestration in vulval cone (outside); E, F: Cyst surface punctations. (Scale bar = 20 µm).

**Figure 4:**
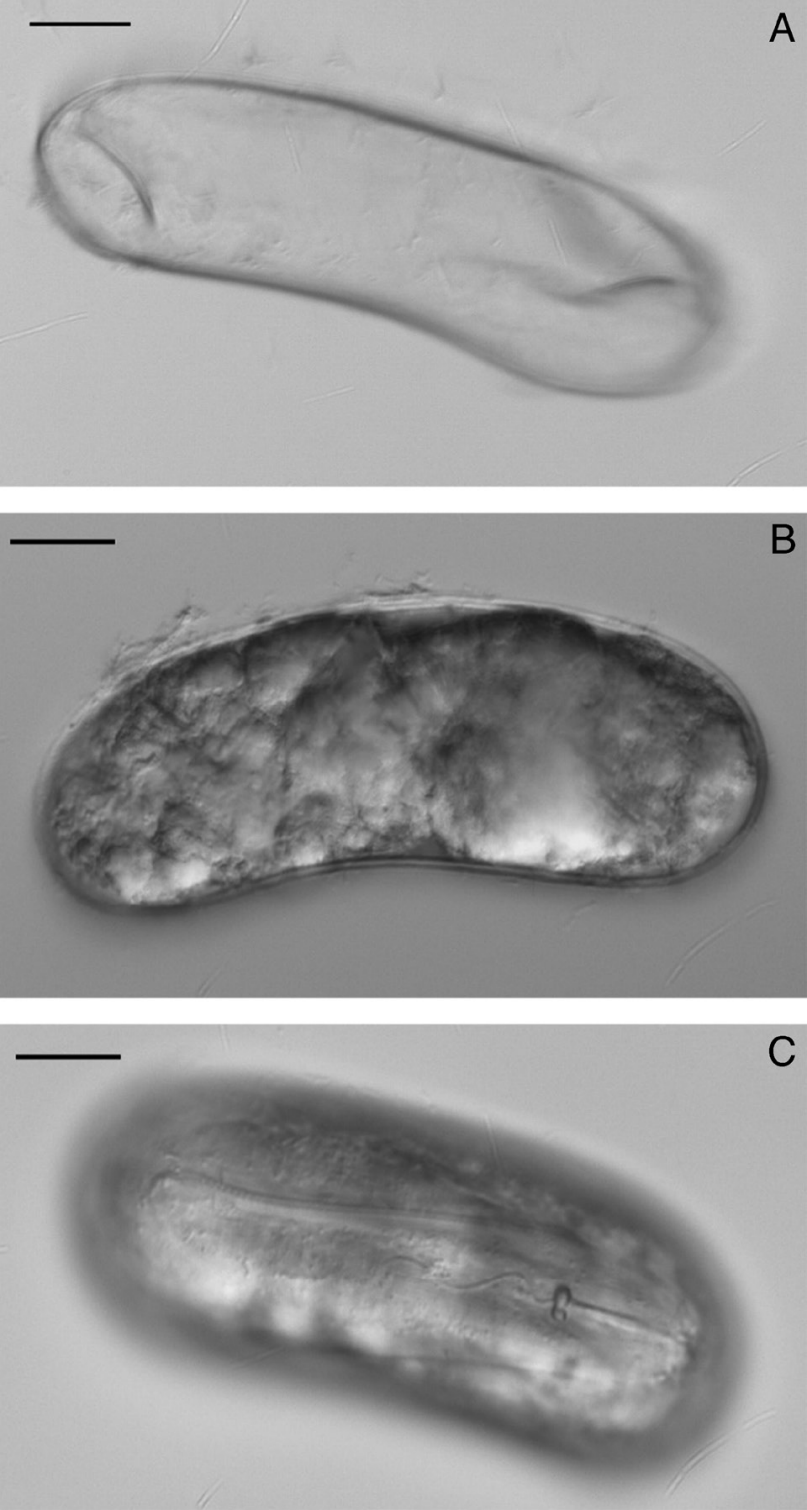
Egg of *C. tianzhuensis* n. sp. A: Smooth eggshell; B: Embryo egg; C: Body of developed J2 in egg. (Scale bar = 20 µm).

**Figure 5:**
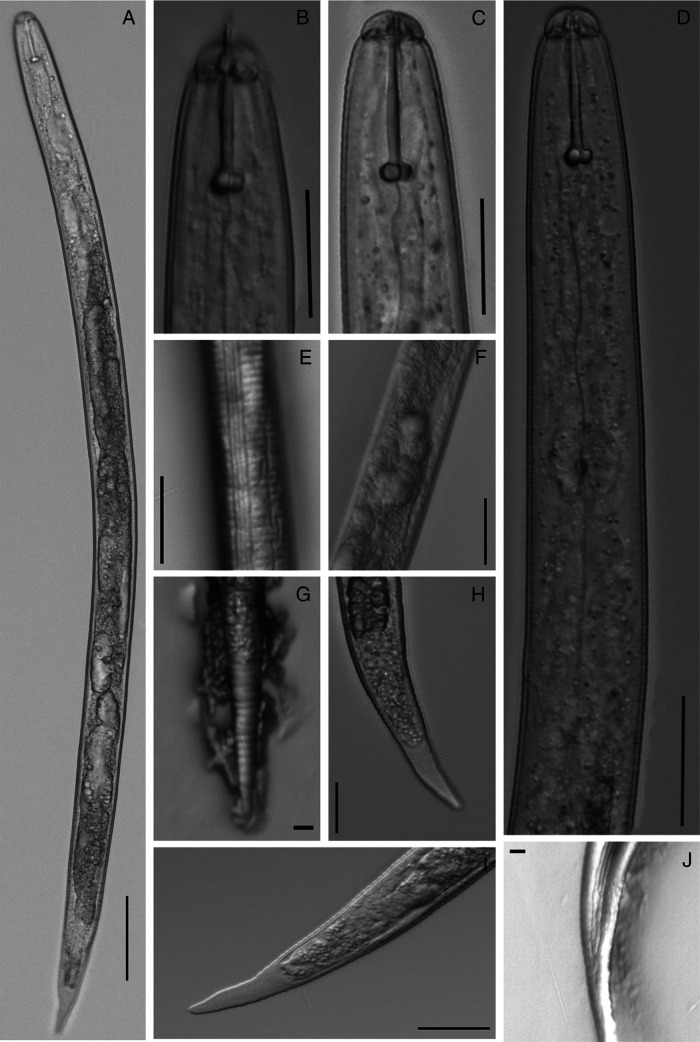
Light micrographs of second stage juvenile of *C. tianzhuensis* n. sp. A: Entire body; B, C: Head region; D: Anterior region of J2; E: Lateral field; F: Genital primordium; G: Annulation of tail; H-I: Tail; J: Lateral field of tail. (Scale bar: A = 50 μm, B, C, D, E, F = 20 μm, H, I = 10 μm, G, J = 5 μm).

Figures 1–5; Measurement Table 1.

**Table 1.** Morphometrics of *C. tianzhuensis* n. sp.

Stage	Character	Holotype	Paratype
Cyst			
	*n*		20
	L (excluding length)	531	571.2 ± 79.1 (511.0–761.0)
	Diam.	429	454.3 ± 56.8 (360.5–558.0)
	L/Diam.	1.23	1.3 ± 0.1 (1.1–1.6)
	Fenestral diam.	22.8	23.4 ± 3.8 (20.0–31.5)
Second-stage juvenile			
	*n*		20
	Body length		538.5 ± 25.0 (494.5–591.5)
	Body width at mid-body		23.2 ± 1.4 (20.0–25.0)
	a		23.4 ± 2.0 (20.6–27.2)
	b		3.7 ± 0.3 (3.1–4.2)
	c		10.0 ± 0.8 (8.4–11.6)
	c′		3.9 ± 0.3 (3.5–4.5)
	Lip region height		4.8 ± 0.4 (4.0–5.0)
	Lip region diam.		10.7 ± 0.8 (9.0–12.0)
	Stylet length		24.9 ± 0.8 (22.5–26.0)
	Stylet base height		2.5 ± 0.3 (2.0–3.0)
	Stylet base width		5.1 ± 0.54 (4.0–5.5)
	Median bulb from anterior end (MB)		77.8 ± 4.2 (69.5–85.0)
	Opening of dorsal pharyngel gland from stylet base (DGO)		4.9 ± 0.5 (4.0–6.5)
	Excretory pore from anterior end (EP)		111.2 ± 6.2 (97.5–124.0)
	Median bulb width (MBW)		11.8 ± 1.1 (9.5–13.5)
	Diam. at anus		13.8 ± 0.5 (11.5–15.0)
	Tail length		54.1 ± 3.6 (46.5–59.0)
	Hyaline portion tail		25.8 ± 2.1 (21.5 –28.5)
	L/MB		6.9 ± 0.5 (6.1–8.0)
	TL/H		2.1 ± 0.2 (1.8–2.4)
Egg			
	*n*		20
	Length		117.9 ± 11.6 (101.5–144.0)
	Width		51.3 ± 4.1 (41.0–61.5)
	Length/Width		2.3 ± 0.3 (1.9–2.9)

Note: All measurements are in μm, and in the form: mean ± standard (range).

### Description

#### Cyst

Cyst body usually lemon-shaped, some rounded, with protruding neck and vulval cone, light brown to black in color (Figures 1C, H, 2A, B). Cyst surface with zigzag pattern, punctations often present, sometimes heavy (Figure 3E, F). Vulval cone top abullate, circumfenestrate (Figures 1E, 3C, D). Vulval denticles usually present, located below upper surface of fenestra, in small clusters, measuring about 8.2 µm in length and 4.5 µm in width (Figure 3A, B). Bullae and underbridge absent. Anus distinct. Cysts containing 90–160 eggs (Figure 2C).

#### Second-stage juvenile

Body slightly curved ventrally after heat treatment, vermiform and tapering anteriorly and posteriorly (Figure 5A). Annulation of body distinct, measuring 1.6 µm wide at mid-body. Lip region offset, measuring 4.0–5.0 (4.8) µm height and 9.0–12.0 (10.7) µm wide. Stylet well developed, knobs rounded and slightly concave anteriorly (Figures 1A, B, 5B, C). Median bulb rounded with strong valvular apparatus, measuring 9.5–13.5 (11.8) µm width. Pharyngeal glands well developed, overlapping ventrally. Hemizoind about one to two annules long, excretory pore situated 97.5–124.0 (111.2) µm from anterior end, one to two annules posterior to hemizonid. Lateral field with four longitudinal incisures forming three bands, averaging 4.1 µm wide at mid-body and each band about 1.0 µm wide (Figures 1D, 5E). Genital primordium situated at 58–63% of body length behind anterior end, with four distinct nucleate cells (Figure 5F). Tail gradually tapering to a finely rounded terminus (Figures 1F, G, 5G–I). Hyaline portion irregularly annulated occupying about 48% of tail length, with V-shaped or U-shaped of outline (Figure 5H, I). Phasmid present (Figure 5J).

#### Eggs

Egg shells without visible markings or punctations, body of developed J2 in eggs folded about five times (Figure 4A–C).

#### Male

Not found.

#### Type material

Holotype cyst, 25 cysts and 25 second-stage juveniles paratypes material were deposited in the nematode collection of the Department of Plant Protection, Biocontrol Engineering Laboratory of Crop Diseases and Pests of Gansu Province, Lanzhou, China.

#### Type host and locality

*Cactodera tianzhuensis* n. sp. was collected from the roots and rhizosphere soil of *Polygonum viviparum* L. (Polygonaceae) in Tianzhu county, Wuwei city, Gansu Province, China (GPS coordinates are: N 37°10′29″; E 102°49′24″). This site located in continental highland with the vegetation type of meadow grassland and the soil is composed of chernozems. The climatic parameters of site include a 450 mm of average rainfall and an approximate −2°C air temperature.

#### Etymology

*Cactodera tianzhuensis* n. sp. is named after the type locality of its isolation.

#### Diagnose and relationships

*Cactodera tianzhuensis* n. sp. is characterized by lemon-shaped or rounded cysts that have protruding necks and vulval cones. The cysts are *ca* 571 (511–761) µm long × 454 (361–558) µm wide and with a circumfenestrate vulval cone, vulval denticle present but bullae and underbridge often absent, anus distinct. Cysts containing 90–160 eggs. Second-stage juveniles are vermiform, slightly curved ventrally and *ca* 539 (495–592) µm long, stylet well-developed with the rounded stylet knobs to slightly concave anteriorly. Lateral field with four incisures. Genital primordium situated at 58–63% of body length. Tail gradually tapering to a finely rounded terminus with *ca* 54 (47–59) µm long, outline of hyaline portion is V-shaped or U-shaped. Egg shells without visible markings or punctations, body of developed J2 in eggs folded about five times.

The new species belongs to the genus *Cactodera*, up to now, the genus *Cactodera* contains seventeen species (including *C. tianzhuensis* n. sp.). These species are similar in circumfenestrate fenestration, without bullae and underbridge, lateral field of J2 with four incisures. Morphologically, *C. tianzhuensis* n. sp. is closest to *C. thornei* with many overlapping morphometrics, such as cyst size, the length of J2 body, DGO, stylet, tail and hyaline tail, but differs from *C. thornei* in fenestral diam. (20–32 vs 31–36 μm) for cyst and eggshell surface (smooth vs punctate). *C. tianzhuensis* n. sp. is similar to *C. cacti*, but differs from this species in the longer DGO (4.0–6.5 vs 2.9–4.4 μm), the longer length of hyaline tail (22–29 vs 14–21 μm) and eggshell surface (smooth vs punctate). In addition, *C. tianzhuensis* n. sp. can be easily distinguished from several species of *Cactodera*, namely *C. chenopodiae*, *C. eremica*, *C. evansi*, *C. galinsogae*, *C. milleri*, *C. rosae* and *C. solani* by eggshell surface smooth vs punctate. It can be distinguished from *C. radicale*, *C. salina* and *C. torreyanae* by vulval denticles present vs absent.

Any other species than mentioned above of the genus *Cactodera*, *C. tianzhuensis* n. sp. can be distinguished from *C. acnidae* by shorter fenestral diam. (20–32 vs 30–33 μm), longer J2 body length (495–592 vs 361–448 μm), DGO (4.0–6.5 vs 2.5–3.0 μm), and tail length (47–59 vs 43–48 μm). It differs from *C. amaranthi* by longer J2 body length (495–592 vs 340–460 μm), stylet length (23–26 vs 20–21 μm), tail length (47–59 vs 43–48 μm), and hyaline tail length (22–29 vs 12–16 μm). *C. tianzhuensis* n. sp. is different from *C. estonica* by a smaller L/W cyst ratio (1.1–1.6 vs 2.0–2.4), longer J2 body length (495–592 vs 426–465 μm), DGO (4.0–6.5 vs 3.4–4.3 μm), tail length (47–59 vs 36–44 μm), and hyaline tail length (22–29 vs 14–21 μm). It differs from *C. wessi* by smaller L/W cyst ratio (1.1–1.6 vs 1.2–2.3), longer J2 body length (495–592 vs 426–465 μm), stylet length (23–26 vs 20–22 μm), tail length (47–59 vs 43–50 μm), and hyaline tail length (22–29 vs 17–24 μm).

In addition, comparative important morphological and morphometric characters of *C. tianzhuensis* n. sp. with sixteen valid species of the genus *Cactodera* are listed in Table 2.

**Table 2.** Morphological and morphometrics of characters of cysts, eggs and J2s, useful for identification of *Cactodera* species.

	Stage											
	Cyst					Second-stage juvenile					Egg	
Species	Length	Width	L/W ratio	Vulval denticles	Fenestral diam.	Length	DGO	Stylet Length	Tail Length	Hyaline tail length	Eggshell surface	Original des.
*C. acnidae*	504–857	319–493	1.2–2.4	–	30 × 33	361–448	2.5–3.0	19–25	43–48	17–26	Smooth	Schuster and Brezina (1979)
*C. amaranthi*	525–774	370–550	1.1–1.7	Present	25–38	340–460	3.9–5.1	20–21	32–40	12–16	Smooth	Golden and Raski (1977)
*C. cacti*	389–658	323–598	1.0–1.4	Present	16–30	456–540	2.9–4.4	24–26	49–60	14–21	Punctate	Graney and Bird (1990)
*C. chenopodiae*	423–585	283–398	1.2–1.7	Absent	20–26	438–539	3.1–4.3	22–26	39–51	17–28	Punctate	Feng et al. (2018)
*C. eremica*	530–810	290–590	1.2–1.9	Absent	14–25	440–510	3.5–6.0	25–28	36–47	17–23	Punctate	Baldwin and Bell (1985)
*C. estonica*	686–1014	312–468	2.0–2.4	Present	18–30	426–465	3.4–4.3	22–24	36–44	14–21	Smooth	Golden and Raski (1977)
*C. evansi*	416–528	284–384	1.2–1.7	–	18–23	358–420	2.8–4.0	20–24	34–44	16–23	Punctate	Cid Del Prado and Rowe (2000)
*C. galinsogae*	453–675	284–508	1.1–1.7	Absent	33–56	358–443	–	19–31	26–45	10–24	Punctate	Soto et al. (2003)
*C. milleri*	550–849	419–598	1.2–1.6	Present	14–18	370–479	3.2–5.1	21–23	37–49	15–21	Punctate	Graney and Bird (1990)
*C. radicale*	553–986	220–626	1.3–2.6	Absent	17–28	467–520	3.6–5.8	20–27	46–60	15–28	Smooth	Subbotin et al. (2010)
*C. rosae*	460–840	280–560	1.2–2.1	Present	10–21	348–472	–	16–26	31–68	4–8	Punctate	Cid Del Prado and Miranda (2008)
*C. salina*	415–742	193–475	1.4–2.2	Absent	20–28	410–514	2.5–4.0	23–25	31–48	10–31	Smooth	Baldwin et al. (1997)
*C. solani*	291–581	204–505	1.2–1.4	Present	20–36	379–511	3.7–6.9	24–27	28–49	12–23	Punctate	Escobar-Avila et al. (2020)
*C. thornei*	485–806	286–581	1.2–1.9	Present	31–36	446–620	5–7	25–28	49–64	23–28	Punctate	Golden and Raski (1977)
***C. tianzhuensis n. sp.***	**511–761**	**361–558**	**1.1–1.6**	**Present**	**20–32**	**495–592**	**4.0–6.5**	**23–26**	**47–59**	**22–29**	**Smooth**	**In this study**
*C. torreyanae*	364–712	92–432	1.4–2.9	Absent	20×26	390–550	2–4	21–23	32–45	16–25	Smooth	Cid Del Prado and Subbotin (2014)
*C. weissi*	524–598	350–394	1.2–2.3	Present	29–38	407–489	4.5–5.6	20–22	43–50	17–24	Smooth	Golden and Raski (1977)

Note: All measurements are in μm, and in the form: mean ± standard (Range).

### Molecular characterization and phylogenetic relationships

#### D2-D3 region of 28S-rDNA

**Figure 6:**
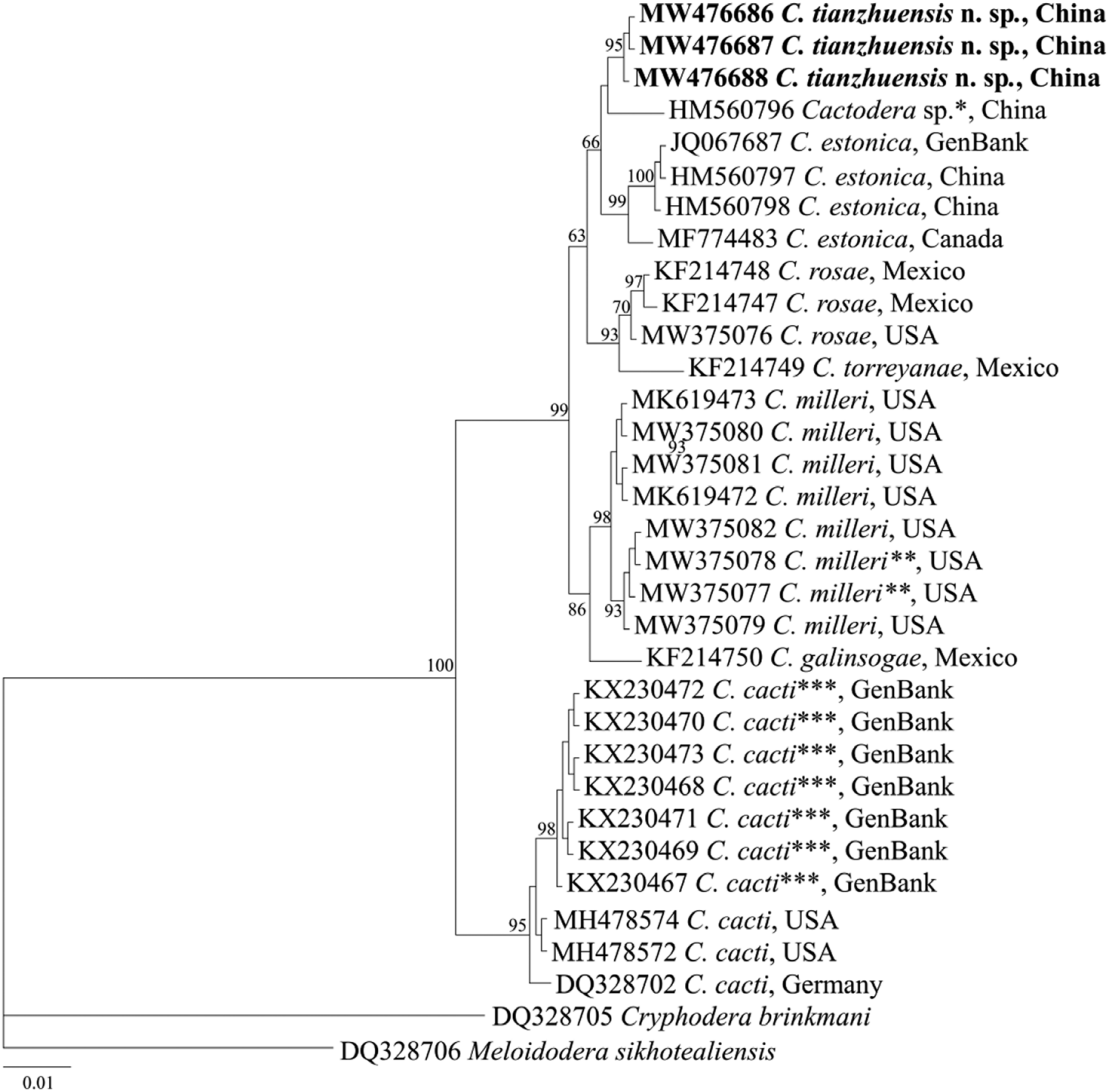
Molecular phylogenetic tree of *C. tianzhuensis* n. sp. (highlighted in bold) inferred from 28 S D2/D3 extension region under GTR + I + G model. The posterior probability values exceeding 50% are given on appropriate clades. *Originally identified as *C. estonica* in the GenBank. **Originally identified as *C. rosae* in the GenBank. ***Originally identified as *C. estonica* in the GenBank.

Three sequences of D2-D3 region (accession no. MW476686-MW476688) from *C. tianzhuensis* n. sp. were obtained without intraspecific sequence variation. The D2-D3 region alignment consisted of 33 ingroup sequences from 8 *Cactodera* species (including the new species and *Cactodera* sp.) and two outgroup sequences from 2 species (*Meloidodera sikhotealiensis*, DQ328706; *Cryphodera brinkmani*, DQ328705). The D2-D3 region sequence similarity between the new species and other *Cactodera* species is as follows: 98.93% (8 bp difference), 98.93–99.20% (6–8 bp difference), 98.93–99.20% (6–8 bp difference), 98.71–98.99% (7–9 bp difference), 98.57% (10 bp difference), 98.15% (13 bp difference) and 95.9–96.68% (24–27 bp difference) for *Cactodera* sp. (HM560796), *C. estonica*, *C. milleri*, *C. rosae*, *C. galinsogae*, *C. torreyanae* and *C. cacti*, respectively. The Bayesian phylogenetic tree of the D2-D3 of 28 S gene under GTR + I + G model (Figure 6) revealed a highly supported (PP = 100) clade of *Cactodera* species, where three sequences of *C. tianzhuensis* n. sp. occupied a basal position. In this tree, *C. tianzhuensis* n. sp. is a sister species of *Cactodera* sp. (HM560736) and *C. estonica* (JQ067687, HM560798, HM560797, and MF774483), which formed a 66% clade, however, the most important morphologically difference between *C. tianzhuensis* n. sp. and *C. estonica* are related to the cyst L/W ratio and J2 length, respectively.

#### ITS-rDNA

**Figure 7:**
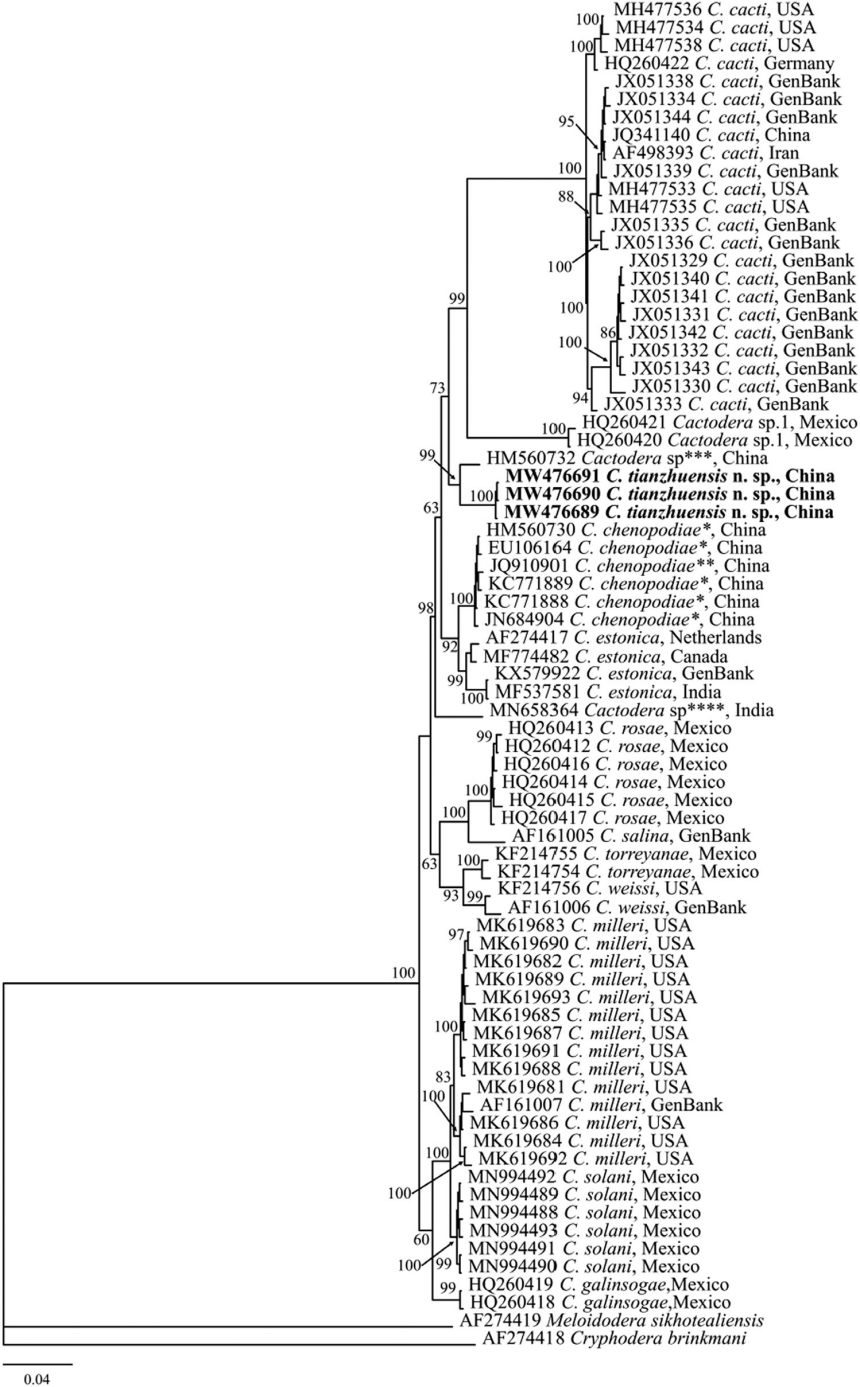
Molecular phylogenetic tree of *C. tianzhuensis* n. sp. (highlighted in bold) inferred from ITS region under GTR + I + G model. The posterior probability values exceeding 50% are given on appropriate clades. *Originally identified as *C. estonica* in the GenBank. **Originally identified as *C. eremica* in the GenBank. ***Originally identified as *C. estonica* in the GenBank. ****Originally identified as *C. estonica* in the GenBank.

Three sequences of ITS region from *C. tianzhuensis* n. sp. (accession no. MW476689-MW476692) were obtained without intraspecific sequence variation. The ITS-rDNA sequence divergences of *C. tianzhuensis* n. sp. showed 30 bp (3.38%), 34–36 bp (3.83–4.05%), 33–38 bp (4.21–4.51%), 43–44 bp (4.89–5.01%), 43–57 bp (4.89–6.48%), 47–49 bp (5.33–5.56%), 47–51 bp (5.33–5.84%), 61–66 bp (6.89–7.46%), 65 bp (7.38%), 91–107 bp (10.5–11.96%) sequence identities with *Cactodera* sp., *C. chenopodiae*, *C. estonica*, *C. solani*, *C. milleri*, *C. torreyanae*, *C. weissi*, *C. galinsogae*, *C. rosae* and *C. cacti*, respectively. ITS region alignment consisted of 73 *Cactodera* sequences from 14 species and two outgroup sequences from 2 species (*Meloidodera sikhotealiniensis*, AF274419; *Cryphodera brinkmani*, AF274418). The Bayesian phylogenetic tree generated from ITS gene under GTR + I + G model is presented in Figure 7, in this tree, all *Cactodera* species formed a 100% clade, *C. tianzhuensis* n. sp. is a sister species of *Cactodera* sp. (HM560732) and cluster together with a well-supported clade (PP = 91), however, it differs from *Cactodera* sp. (HM560732) by 30 bp (3.38%).

## Discussion

Initially, Mulvey (1972) divided several cyst-forming nematodes into five groups based on cyst characteristics. Four species (namely, *H. betulae* Hirschmann and Riggs, 1969; *H. cacti* Filipjev and Schuurmans Stekhoven, 1941; *H. estonica* Kirjanova and Krall, 1963 and *H. weissi* Steiner, 1949) were characterized by cysts with circumfenestrate vulval cone and posterior protuberance in group two. Subsequently, Krall and Krall (1978) proposed the genus *Cactodera* from the type species name and several *Heterodera* species added in this genus. The key morphological characters of the genus *Cactodera* include cysts lemon-shaped to spherical with posterior protuberance, fenestra circumfenestrate, absence of bullae and underbridge, vulval denticles usually present and anus without fenestration, second-stage juveniles have strong stylet, lateral field with four lines and phasmid opening punctiform. Eggshells punctate or smooth (Subbotin et al., 2010). These characteristics clearly indicate that the new species belongs to the genus *Cactodera*. Morphologically and morphometrically, *C. tianzhuensis* n. sp. is most similar to *C. thornei* in having a longer body (average length > 500 μm) and longer tail (average length > 54 μm) that can be differentiated from other *Cactodera* spp. However, the lower and upper morphometric data of the ranges may overlap with other related species, *Cactodera* spp. will be identified more accurately based on morphological, morphometric, and molecular data.

In our molecular phylogenetic studies, *C. tianzhuensis* n. sp. formed a single clade with *Cactodera* species and showed closely related to *C. estonica* and *Cactodera* sp. (original identified in GenBank as *C. estonica*; unpublished). However, sequence divergence (ranged from 6 to 8 bp for 28 S; 30 to 38 bp for ITS) and morphological characteristics can easily distinguish these species. Presently, out of seventeen valid species, six *Cactodera* species are not represented in GenBank database (i.e., *C. acnidae* (Schuster and Brezina, 1979) Wouts, 1985, *C. amaranthi* (Stoyanov, 1979) Krall and Krall, 1978, *C. eremica* Baldwin and Bell, 1985, *C. evansi* Cid Del Prado and Rowe, 2000, *C. radicale* Chizhov, Udalova and Nasonova, 2008, *C. thornei* (Golden and Raski, 1977) Mulvey and Golden, 1983). Thus, sequences information with the genus *Cactodera* is still limited in molecular data and need to be completed in more studies.

*C. tianzhuensis* n. sp. is isolated from *Polygonum viviparum* L. in Tianzhu county, this habitat located in continental highland with the vegetation type of meadow grassland and the soil is composed of chernozems. The previous studies reported only three species of cyst-forming nematodes (two *Heterodera* species and one *Globodera* species) from this habitat. Li et al., 2020 described a new *Heterodera* species found in the rhizosphere of *Microula sikkimensis* and named *Heterodera microulae* (Li, et al., 2020) and several scholars reported *Heterodera avenae* Wollenweber, 1924 associated with meadow grass (*Kobresia myoscuroides*, *Kobresia humilis* and *Achnatherum inebrains*) (Li et al., 2015; Zhang et al., 2019) and *Globodera artemisiae* parasitizing on Chinese herbal medicine (*Artemisia argyi*) (Han et al., 2020). To our best knowledge, there is no report of the genus *Cactodera* damage plants in this habitat and this is first species described of *Cactodera* species in this habitat, the fourth *Cactodera* species in China. Though few studies on the host-suitability of several species of *Cactodera* spp. have been evaluated, barley (*Hordeum vulgare* L.) is known as being a host for *C. galinsogae* and *C. rosae* (Cid Del Prado and Miranda, 2008), and recently described *C. solani* on tomato (*Solanum lycopersicum*) was reported (Escobar-Avila et al., 2020). In addition, Graney and Bird (1990) performed a host range test of *C. milleri* including 34 plant species and indicated this species can reproduce on quinoa (*Chenopodium quinoa*). Moreover, three species of *Cactodera* (namely, *C. chenopodiae*, *C. torreyanae*, *C. solani*) were shown to be endoparasitic to semi-endoparasitic in sessile habit, a characteristic that the juveniles penetrate with anterior body into the host roots and the posterior body protruding from the surface of the roots (Cid Del Prado and Subbotin, 2014; Escobar-Avila et al., 2020; Feng et al., 2018). Therefore, the biology, host-suitability, and distribution of *Cactodera* species (including *C. tianzhuensis* n. sp.) should further studies to explore.

### Key to species of *Cactodera*

(Modified from Cid Del Prado and Subbotin, 2014; Feng et al., 2018; Subbotin et al., 2010)

1. Cyst generally two times or more longer than wide, mean L/W ratio = 2.3...........…*C. estonica*

- Cyst usually less than twice as long as wide, mean L/W ratio = 1.1–1.8…....................…….2

1. Eggshell punctate…........................................3

- Eggshell smooth…........................................11

1. Mean stylet length of J2s ≥ 26 μm…......……. 4

- Mean stylet length of J2s < 26 μm…….….......5

1. J2s tail length = 48–64 μm, hyaline region = 23–28 μm, fenestral diam. = 23–41 μm……*C. thornei*

- J2s tail length = 37–48 μm, hyaline region = 17–24 μm, fenestral diam. = 14–25 μm..…*C. eremica*

1. Mean J2s body length ≥ 411 μm…..………….6

- Mean J2s body length < 411 μm…..…………...9

1. Mean J2s tail length > 40 μm, mean cyst length > 440 μm, mean cyst width > 325 μm….………7

- Mean J2s tail length ≤ 40 μm, mean cyst length ≤ 440 μm, mean cyst width ≤ 325 μm…..*C. solani*

1. b ratio < 3.5, fenestral diam.< 20 μm........*C. milleri*

- b ratio > 3.5, fenestral diam. > 20 μm…..………8

1. Female L/W ratio < 1.4, mean hyaline region of J2s < 22 μm…................................……*C. cacti*

- Female L/W ratio ≥ 1.4, mean hyaline region of J2s ≥ 22 μm……...................…*C. chenopodiae*

1. Fenestral diam. < 25 μm…..…………………. 10

- Fenestral diam. ≥ 25 μm……….…*C. galinsogae*

1. Hyaline region of J2s = 4–8 μm…......…*C. rosae*

- Hyaline region of J2s = 16–23 μm….…*C. evansi*

1. Mean J2s tail length < 40 μm………………....11

- Mean J2s tail length ≥ 40 μm……………........14

1. Mean J2s body length < 406 μm, mean hyaline region < 16 μm…..…………………*C. amaranthi*

- Mean J2s body length ≥ 406 μm, mean hyaline region ≥ 16 μm…………................................13

1. Cyst with distinct vulval cone, J2s stylet length = 21.0–23.0 μm…..........….*C. torreyanae*

- Cyst without distinct vulval cone, J2s stylet length = 23.4–25.0 μm…...................…*C. salina*

1. J2s stylet knobs anterior surface concave, DGO = 4.5–5.6 μm…………….......................15

- J2s stylet knobs anterior surface convex, DGO = 2.5–3.0 μm……………......... *C. acnidae*

1. Vulval denticles present……………………….16

- Vulval denticles absent.……………. *C. radicale*

1. Mean J2s body length < 489 μm, J2s stylet length = 20–22 μm……..............….…. *C. weissi*

- Mean J2s body length ≥ 489 μm, J2s stylet length = 23–26 μm…….…*C. tianzhuensis* n. sp.
